# Research on the Mechanical Properties and Temperature Compensation of an Intelligent Pot Bearing for a Pipe-Type Welding Strain Gauge

**DOI:** 10.3390/s23249648

**Published:** 2023-12-06

**Authors:** Nianchun Deng, Haitang Zhang, Feng Ning, Zhiyu Tang

**Affiliations:** 1 College of Civil Engineering and Architecture, Guangxi University, Nanning 530004, China; dengnch@gxu.edu.cn (N.D.); zhanghaitangwy@163.com (H.Z.); nfeng@cccc4.com (F.N.); 2 Key Laboratory of Disaster Prevention and Structural Safety of Ministry of Education, Guangxi University, Nanning 530004, China

**Keywords:** intelligent bridge bearing, finite element analysis of bearing, welding strain gauge, bearing calibration, temperature compensation

## Abstract

As an important component connecting the upper and lower structures of a bridge, bridge bearings can reliably transfer vertical and horizontal loads to a foundation. Bearing capacity needs to be monitored during construction and maintenance. To create an intelligent pot bearing, a portable small spot welding machine is used to weld pipe-type welding strain gauges to the pot bearing to measure strain and force values. The research contents of this paper include the finite element analysis of a basin bearing, optimal arrangement of welding strain gauges, calibration testing, and temperature compensation testing of the intelligent basin bearing of the welding strain gauges. Polynomial fitting is used for the fitting and analysis of test data. The results indicate that the developed intelligent pot bearing has a high-precision force measurement function and that after temperature compensation, the measurement error is within 1.8%. The intelligent pot bearing has a low production cost, and the pipe-type welding strain gauges can be conveniently replaced. The novelty is that the bearing adopts a robust pipe-type welding strain gauge and that automatic temperature compensation is used. Therefore, the research results have excellent engineering application value.

## 1. Introduction

A bridge bearing constitutes a hinge between the superstructure and substructure of a bridge, which can reliably transfer static and dynamic loads to the ground. Due to the limited space around the bearing, it is difficult to detect potential damage, such as voids, displacements, and reaction forces that exceed the permissible limits. Previously, bearing inspection has mainly relied on visual observations with easy-to-use equipment. Nevertheless, this inspection method is time-consuming and labor-intensive. Therefore, it is of great engineering application value to develop an intelligent device that can monitor the bearing performance and condition in real time over the long term [1,2,3,4].

In recent years, engineers have focused their attention on the research and development of intelligent bearings. For example, Schulz et al. [5] exploited a multiaxis strain sensor based on a double-overlapping grating with significant advantages, including polarization selectivity. The sensor was configured as a load cell with an embedded distributed transverse strain sensor. This load cell is capable of measuring transverse strains and strain gradients. Agrawal et al. [6] developed a pressure sensor intelligent bearing system for elastic bearings, which included an embedded multiaxis fiber grating strain sensor, tactile pressure sensor, and LVDT measuring instrument. Cho et al. [3] proposed the design concept of a load-measuring pot bearing with a built-in small-size button-type load cell inserted in the base plate of the bearing and experimentally examined its measurement accuracy and the possibility of achieving dynamic measurements. Chang et al. [7,8] overcame the replacement of sensors and introduced a built-in piezoelectric composite generating element (PCGE) in the intelligent bridge bearing based on FBG sensors to determine where PCGE provides the most efficient power generation. Xiong et al. [9,10,11,12,13] added a fiber grating force measurement module to the bearing to create a force-measuring bearing. Through a standard press, the vertical bearing capacity of the approach was applied to the bearing, the corresponding optical fiber grating wavelength value was read, and the linear function relationship between the force value and the wavelength value was obtained to realize the detection function of the vertical bearing capacity. However, there have been no studies on temperature compensation, and there is a high risk of the sensor falling off due to adhesive aging. The hydraulic force sensor is a device made of special steel in a closed cavity and filled with hydraulic oil. When subjected to pressure stress, it can sense stress and convert pressure signals into current signals through a pressure transmitter, which is convenient for signal collection and transmission. Xiong et al. [14,15,16] studied an embedded fluid pressure sensor in a bearing, which was made into an intelligent bearing. However, once the hydraulic pressure entered the steel, the data drifted. Liu et al. [17] and Hang et al. [18] showed that based on the original structure and mechanical properties of a pot bearing, the internal stress distribution of the bearing was measured by built-in flexible thin-film pressure sensors. The pressure resistance varied with pressure, but the film material was very sensitive to changes in temperature and was prone to signal drift and hysteresis.

In this study, based on the analysis of the original structure and mechanical properties of traditional pot bearings, the support is simulated and analyzed to determine the best spot welding arrangement method for spot welding sensors on the support. Then, the vertical bearing capacity of the support is calibrated under normal temperature to obtain the calibrated fitting curve of the spot welding sensor bearing, and then the temperature compensation test of the support is conducted. The measurement error caused by thermal zero drift and thermal sensitivity drift of the sensor is corrected, and then the support angle test is carried out.

## 2. Finite Element Analysis of Supports

**Figure 1:**
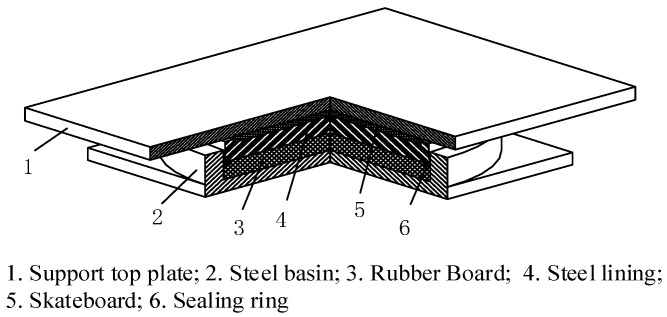
Structural diagram of a basin-type rubber bearing.

The research focus of this work is based on a GPZ (II) 2 SX type bidirectional basin rubber bearing. The components of the structure are composed of six parts, namely a support roof, a steel basin, a rubber plate, a steel lining plate, a slide plate, and a sealing ring, as shown in Figure 1. The total height of the bearing is 82 mm; the size of the steel basin bottom is 15 mm; the thickness of the basin ring is 27.5 mm; the height of the pressure-bearing rubber plate is 21 mm; the height of the steel lining plate is 21 mm; the size of the PTFE slide plate is 7 mm; and the height of the stainless steel slide plate is 7 mm. The thickness of the slide plate is 4 mm, and the top plate height is 16 mm.

A finite element simulation model is set up to explore the optimal arrangement of the sensors on the support. The support model is established by the parametric design language APDL in ANSYS 2022 R2 software, which can effectively perform repeated analyses of a model that must be modified many times. The sealing ring and the bolts, which are not key research objects, are not involved in the modeling analysis.

**Figure 2:**
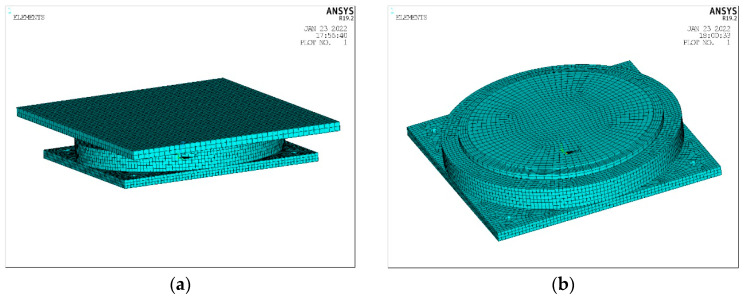
Finite element model of the basin rubber bearing. (**a**) All of finite element model; (**b**) Segment of finite element model.

The contact analysis of ANSYS is nonlinear. During the solution process, the stiffness mutation might render the total stiffness matrix ill-conditioned, making it difficult to reach convergence. The penalty function method and the Lagrange multiplier method are used in this analysis. The mixed-use extended Lagrange method is less sensitive to contact stiffness and relatively easy to converge [19,20,21]. The contact stiffness FKN is taken as 0.8, and the contact intrusion tolerance coefficient is assumed to be 0.1. In the finite element model, as shown in Figure 2, SOLID45 elements are used for the support top plate, steel basin, steel lining plate, and stainless steel sliding plate; SOLID185 elements are used for the pressure-bearing rubber and the PTFE sliding plate. This element has displacement and mixed interpolation modes, which are almost impossible to simulate. The hyperelastic constitutive model is used for compressed elastoplastic materials and completely incompressible hyperelastic materials, which adopts the third-order Ogden model with optimal stress–strain behavior. For this model, the strain level can reach 700%. The parameters of the Ogden model are given in Table 1. In the contact analysis, the surface–surface contact method is adopted. The contact unit is conta173, and the target unit is targe170; the friction coefficients between the steel basin, the bottom surface of the intermediate steel plate, and the pressure-bearing rubber plate are all taken to be equal to 0.8; the friction coefficient between the bottom surface of the PTFE plate and the intermediate steel plate is also assumed to be 0.08; the friction coefficient between the top surface of the PTFE plate and the stainless steel plate is considered to be 0.03; the friction coefficient of the transverse slider is taken as 0.03; and all contact surfaces are defined as a standard contact, that is, a one-way contact. The material parameters of the bearing are listed in Table 2. The established finite element model of the bearing is shown in Figure 2.

**Figure 3:**
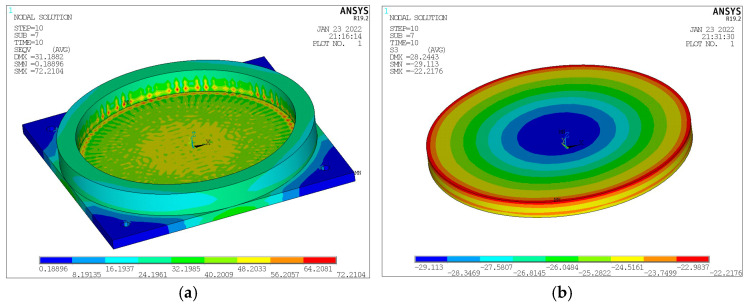

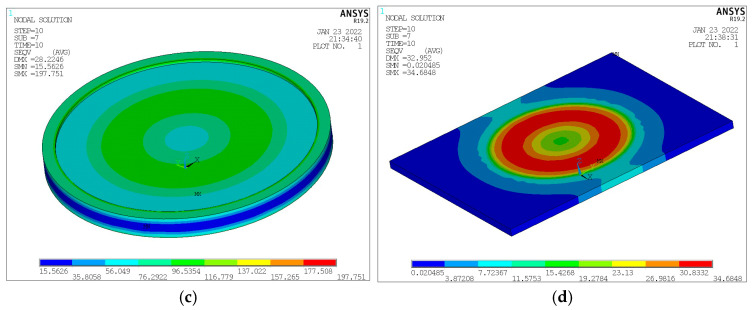
Finite element output. (**a**) von Mises stress of the steel basin; (**b**) Main compressive stress of the rubber plate; (**c**) von Mises stress of the steel lining plate; (**d**) von Mises stress of the top plate.

A vertical design load of 2 MN is applied at the top plate of the finite element model bearing. After the finite element iterative calculation, the stresses of each bearing component are obtained, as shown in Figure 3. From the von Mises stress cloud diagram of the steel basin, illustrated in Figure 3a, the maximum stress occurs at the junction of the basin ring and the basin bottom. Figure 3b presents the pressure-bearing rubber sheet’s principal compressive stress cloud diagram with a uniform stress distribution under the three-way compression state. This law is in line with the characteristics of hyperelastic materials. Figure 3c shows the von Mises stress cloud diagram of the steel lining plate, where the stress in the central part of the steel lining plate is relatively large, up to 198 MPa, but it is also within the allowable stress range of the material. The stress of the outermost ring is very small due to the fact that the diameter of the polyvinyl fluoride sliding plate is smaller than that of the steel lining plate, so the pressure on the outermost ring of the steel lining plate is very small. According to the stress cloud diagram of Figure 3d, the top plate von Mises stress changes from the center to the periphery, first increasing and then decreasing, ranging between 3 MPa and 35 MPa.

## 3. Pipe-Type Welding Strain Gauge Layout

**Figure 4:**
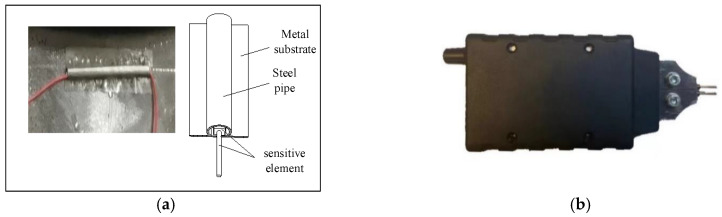
Welding strain gauge and portable spot welding machine. (**a**) Welding strain gauge; (**b**) Portable spot welding machine.

For the pipe-type welding strain gauge, the steel pipe and the metal substrate are joined. The structure has sensitive components (resistance strain gauges) and adhesive. Then, the pipe-type spot welding strain gauges are welded to the metal. Using a portable spot welding machine, the pipe is spot-welded to the steel plate. This method generates a small current during spot welding, with a voltage of only 3 V to 5 V, which will not damage the internal structure. Figure 4a shows the welding strain gauge on the component of the bearing. Figure 4b shows a portable spot welding machine.

**Figure 5:**
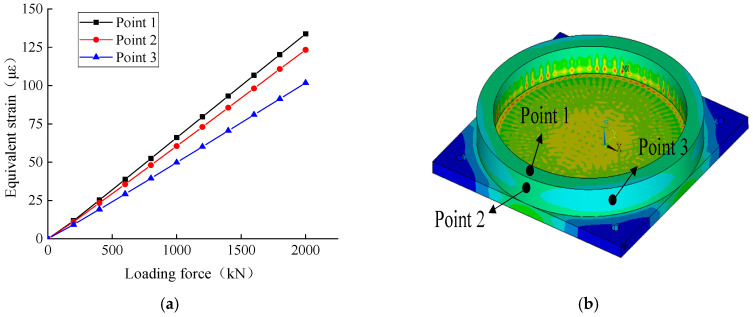
Equivalent stress variation curve of basin ring measuring points with different loading force values. (**a**) Stress variation curve; (**b**) stress cloud diagrams.

Based on the stress cloud diagrams, it is likely that the stress state of the steel basin of the support is the best. Thus, this position is the most suitable for installing welding strain gauges. Appropriate measuring points are selected on the support steel pot to confirm the most suitable welding strain gauge installation position on the steel pot, and strain data are extracted for analysis. Hence, the strain values of 3 measuring points are collected, and the loading force value-equivalent strain curve is depicted, as shown in Figure 5. Notably, the strain values of the three measuring points follow the sequence: measuring point 1 > measuring point 2 > measuring point 3. However, the difference between these three points is small.

The welding strain gauge welding equipment contains an energy storage capacitor spot welding machine, which melts the welding strain gauge substrate and the metal component to be measured by instantaneous high current. The welding procedure of the strain gauge is as follows. First, the substrate of the welding strain gauge and the component to be measured are cleaned. Subsequently, the substrate is flattened on the component to be measured along the measurement direction, and then a spot welder is used to weld 4 points on the four corners of the substrate. Finally, at least two rows of solder joints are welded on both sides of the substrate along the measurement direction, while the distance between the solder joints is between 0.8 and 1.5. Zhang [23] and Shen et al. [24] found that this arrangement ensures high connection strength.

**Figure 6:**
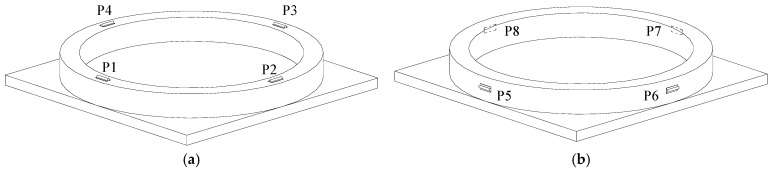
Schematic diagram of the steel basin welding strain gauge layouts. (**a**) 1st layout; (**b**) 2nd layout.

According to the diagrams of Figure 5, measuring point 3 presents the smallest strains and can be discarded. Therefore, two layouts of the welding strain gauge positions are assumed based on measuring points 1 and 2, as shown in Figure 6a,b.

## 4. Calibration Test of the Vertical Bearing Capacity of the Intelligent Support for Welding Strain Gauges

A calibration test is carried out to realize the force measurement function of the support on the points where the welding strain gauge is arranged. First, the strain-loading force curve of the intelligent support for welding strain gauges under normal temperature conditions is obtained. Then, a temperature compensation test of the support is carried out, and the relationship above the curve is corrected, accounting for the temperature to improve the measurement accuracy of the intelligent support.

### 4.1. Calibration and Temperature Compensation Fitting Method

Polynomial, piecewise, and cubic spline fittings are commonly used in the sensor data fitting method. In this paper, these methods are combined to fit and analyze the test data of the force measurements of the bearing.

In the actual loading experiment process, the sensor signal curve regularity is inconsistent when the loading force is very small and when it exceeds a certain force value. The fitting method is used to divide the curve into n segments for polynomial fitting according to the trend of the data curve. The segmented polynomial is represented by (1):

(1) $$y_{i}=a_{i}+b_{i}x+c_{i}x^{2}+d_{i}x^{3}\;\;\;x_{n}\leq x\leq x_{n+1}$$

where $y_{i}$ represents the polynomial equation of the i segment. The fitting aims to find a m degree polynomial for the i segment data $\left(x_{k},y_{k}\right),k=1,2,\ldots ,t$.

A spline is a piecewise polynomial function, and the expression for a k-order (k−1) B-spline is:

(2) $$C(u)=\sum_{i=0}^{n}P_{i}N_{ik}\left(u\right)$$

The corresponding cubic B-spline curve k=4 is given by the following equation:

(3) $$C(u)=\sum_{i=0}^{n}P_{i}N_{i4}\left(u\right)$$

Lv [25] proved that the above formulae $P_{i}$ define the coordinate values of the n+1 vertices of the characteristic polygon. $N_{i4}\left(u\right)$ represents the harmonic function of the fourth order, that is, the third stage of the cubic B-spline, also known as the basis function, and n is the number of vertices of the characteristic polygon.

According to the recurrence formula, the cubic B-spline harmonic function can be defined as follows:

(4) $$N_{i1}\left(u\right)=\left\{ \begin{array}{l} 1\;\;\;\mathrm{when}\;t_{1}\leq u\leq t_{i+1}\\ 0\;\;\;\;\mathrm{other} \end{array}\right.$$

(5) $$N_{ik}\left(u\right)=\frac{\left(u-t_{i}\right)N_{ik-1}\left(u\right)}{t_{i+k-1}-t_{i}}+\frac{\left(t_{i+k}-u\right)N_{i+1,k-1}\left(u\right)}{t_{i+k}-t_{i+1}}\;\;\left(k=4,3,2,1\right)$$

When the denominator is zero, the value that defines the fraction is zero.

The above equations $t_{i}$ represent the control point node value, which controls the shape of the curve, and the node value ranges from $t_{0}$ to $t_{n+4}$. The nonclosed curve is used in this system. Therefore, the $t_{i}$ value rules are as follows:

(6) $$t_{i}\left\{ \begin{array}{ll} 0 & i<4\\ i-3 & 4\leq i<n\\ n-2 & i>n \end{array}\right.$$

Thus, the values $t_{i}$ are: (0,0,0,0,1,2,…,n−3,n−2,n−2,n−2,n−2,).

### 4.2. Calibration Test of the Vertical Bearing Capacity at Normal Temperature

**Figure 7:**
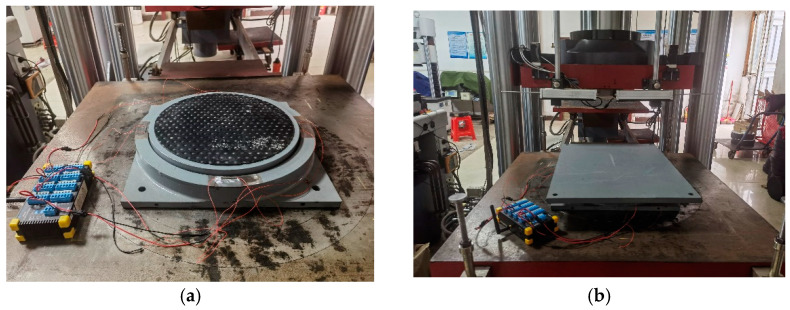
Test loading device. (**a**) CZLYB-4B pot bearing; (**b**) test loading device.

The loading support compression shear test machine and GPZ-II pot bearing are tested in the testing center room of Liuzhou OVM Machinery Co., Ltd. (Liuzhou, China), as shown in Figure 7. The test loading steps are as follows:

Step 1: Install the test support and equipment, and connect the data acquisition instrument.

Step 2: Preload. The load increases to 2000 kN at a speed of 2.0 kN/s; it remains constant for two, and the specimen is unloaded. Three cycles of loading are performed.

Step 3: Gradual loading. There are seven loading levels (0 kN, 200 kN, 400 kN, 800 kN, 1200 kN, 1600 kN, and 2000 kN) in total, and the loading speed of each level is 2.0 kN/s. The specimen is unloaded after the last level of loading is completed. The procedure is repeated three times.

**Figure 8:**
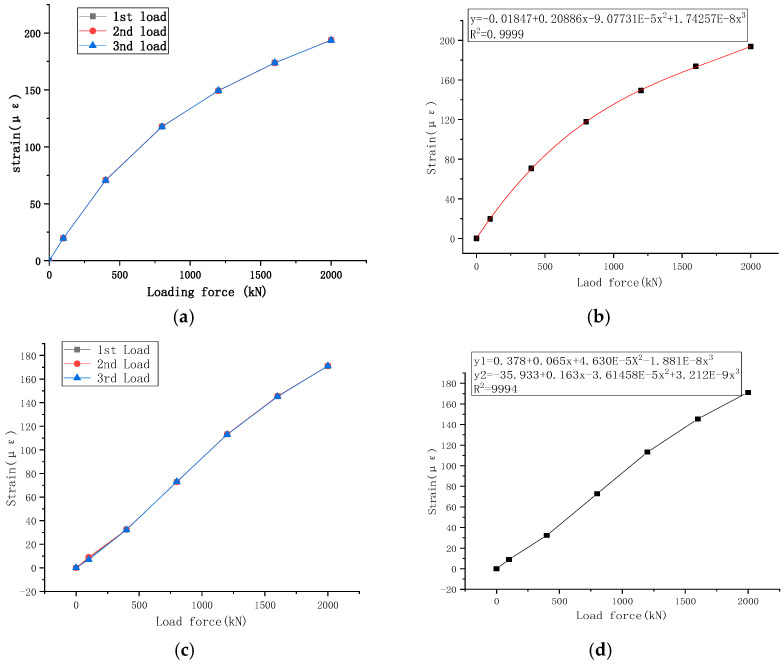
Finite element output curve. (**a**) Mean strain variation curve of basin ring P1~P4; (**b**) linear fitting of P1~P4 strain mean of the basin ring; (**c**) mean strain variation curve of basin ring P5~P8; (**d**) linear fitting of P5~P8 strain mean of the basin ring.

The data obtained from three loading cycles on the intelligent basin support of the welding strain gauge are listed in Table 3. The welding strain gauges P1~P4 and P5~P8 of the steel basin are arranged symmetrically, and the average value of their strains is used for analysis. As shown in Figure 8a,c, the strain change curves of the welding strain gauge for three loading cycles are plotted for the two layouts. The strain change points of the welding strain gauge at points P1 to P4 of the basin support in Figure 8b are formed into small intervals and fitted as straight lines with input signals as independent variables. The cubic spline curve interpolation method is smooth and stable in the interpolation function curve and can form a continuous smooth curve from discrete measurement points. The cubic polynomial fitting method is as follows:

(7) $$y=-0.01847+0.020886x-9.07731\times 10^{-5}x^{2}+1.74257\times 10^{-8}x^{3}$$

The goodness of fit R^2^ is greater than 0.9999.

From Figure 8d, the strain change points of the P5~P8 welding strain gauges for the pot-type support are obtained by using a method of cubic spline curve interpolation and fitting with two segments of cubic polynomials.

(8) $$\left\{\begin{array}{c} y_{1}=0.378+0.065x+4.63\times 10^{-5}x^{2}-1.881\times 10^{-8}x^{3}\\ y_{2}=-35.933+0.165x-3.61458\times 10^{-5}x^{2}+3.212\times 10^{-9}x^{3} \end{array}\right.$$

The goodness of fit R^2^ is greater than 0.9994.

### 4.3. Temperature Compensation Test

In practice, the sensitive elements of most sensors are made of semiconductors, which use the piezoresistive effect to sense changes in stress. Zhang [26] found that semiconductors are highly susceptible to ambient temperature variations due to their material properties, which make the sensors prone to thermal zero drift and thermal sensitivity drift, which lead to a decrease in measurement accuracy.

**Figure 9:**
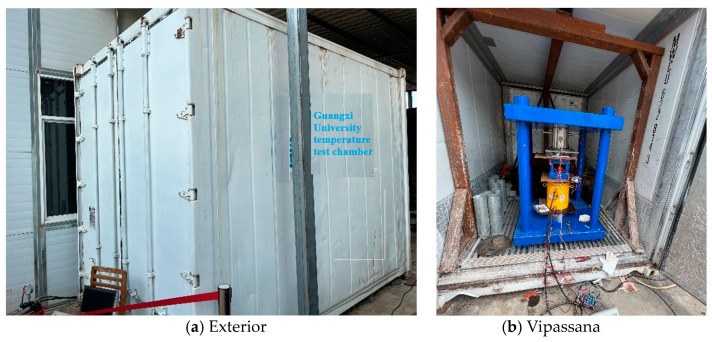
Temperature test box and Vipassana.

Hence, it is necessary to conduct a temperature compensation test of the sensor to define the corresponding temperature sensitivity coefficient of the sensor. This test is carried out in a temperature test box with a loading device, as shown in Figure 9.

**Figure 10:**
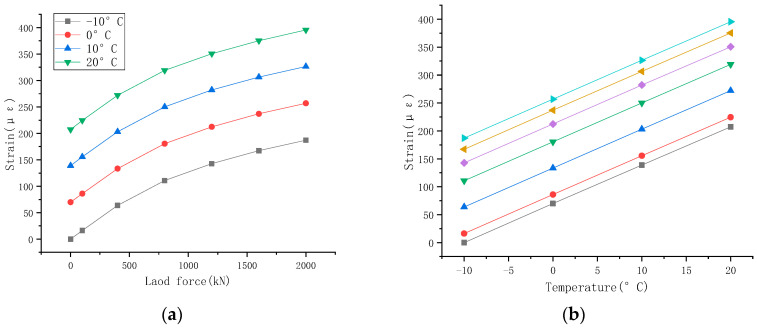

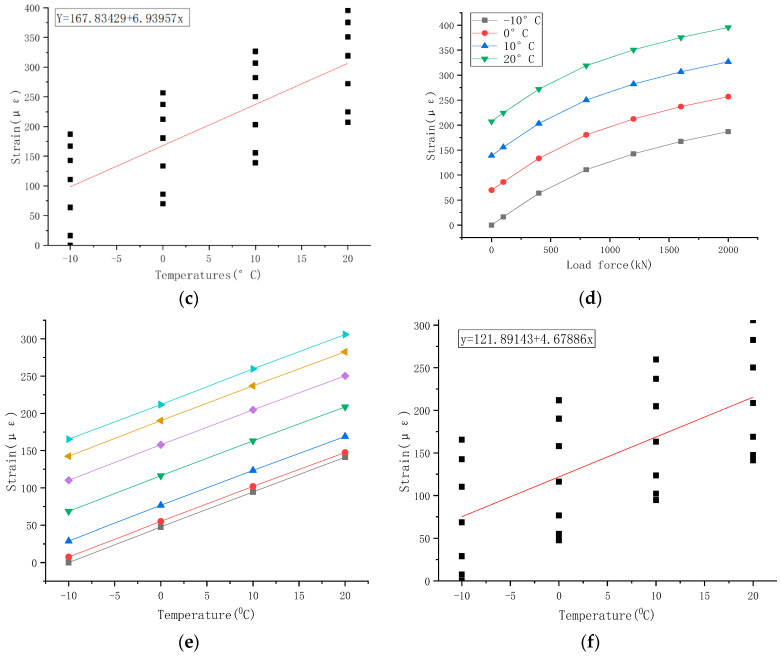
Finite element output curve. (**a**) Variation curve of the mean strain value of the basin ring P1~P4 at different temperatures; (**b**) relationship between strain and temperature at P1~P4; (**c**) fitting the relationship between strain and temperature at P1~P4; (**d**) variation curve of the mean strain and temperature at P1~P4 at different temperatures; (**e**) relationship between strain and temperature at P5~P8; (**f**) fitting the relationship between strain and temperature at P5~P8.

The test procedure is identical to the vertical bearing capacity calibration test at room temperature, as described in Section 4. From top to bottom, the components of the device are a pressure ring, a steel backing plate, a support, a steel backing plate, a heightening block, a steel backing plate, jack, a steel backing plate, and a heightening block. The pressure ring model is CZLYB-4B, the indication repeatability R ≤ 0.3%FS, and the indication error δ ≤ 0.3%FS. The test temperature is divided into four levels: −10 °C, 0 °C, 10 °C, and 20 °C. Each time the specified temperature is reached, it is kept constant for 1 h during loading. The data obtained from the welding strain gauges applied to the intelligent support during the test are presented in Table 4. The strain change curves recorded by the welding strain gauges at different support positions under different temperatures are drawn in Figure 10a,d for the two layouts. As the temperature increases, the curve shifts upwards, and the strain gradually increases. Taking −10 °C as a reference, the average value of the increase in strain is calculated at each temperature level. Then, a scatter diagram of the increase in strain of the welding strain gauge is drawn at each position under different temperatures, as shown in Figure 10b,e. The least squares method was applied for analysis, and the linear fitted curves are shown in Figure 10c.

(9) y=167.83429+6.93957x

Therefore, this formula represents the slope when the temperature compensation coefficient α is 6.93957. Equation (7) compensates for when the temperature compensation equation is $x=x_{c}-\alpha (T_{c}-T_{0})$. The value of $x_{c}$ is a measurement value. The values of $T_{c}$ are the measurement temperature values. The values of $T_{0}$ are the standard temperature values.

The linear fitted curve is shown in Figure 10f.

(10) y=121.89143+4.67886x

Thus, this formula represents the slope when the temperature compensation coefficient α is 4.67886. Equation (8) compensates for when the temperature compensation equation is $x=x_{c}-\alpha (T_{c}-T_{0})$.

The goodness of fit is the relationship between the welding strain gauge strain and temperature; it can be expressed by polynomials and the welding strain. The temperature sensitivity coefficient of the meter is represented by the slope of the fitted curve, which is the first derivative of the polynomial.

### 4.4. Error Analysis

To analyze the measurement accuracy and verify the feasibility of the intelligent support bearing welding strain gauges presented in this work, force measurement tests were carried out to analyze the measurement accuracy and verify the feasibility of the support at different temperatures. The comparison results, both with and without temperature compensation, are presented in Table 5 and Table 6 for the two layouts, respectively. When temperature compensation is not considered, the measurement error of the welding strain gauge is relatively large, especially when the loading force is small. However, the average error of post-measurement, considering temperature compensation, can be effectively controlled within ±1.8%, leading to high measurement accuracy.

## 5. Support Corner Test

To further test the measurement performance of the intelligent pot bearing with welding strain gauges, a rotation angle test of the horizontal plane of the bearing is carried out. Due to space limitations, only the welding strain gauges at points P1~P4 of the steel basin are analyzed, as shown in Table 7. The testing procedures of the welding strain gauges P5~P8 on the steel basin are the same as those of P1~P4.

**Figure 11:**
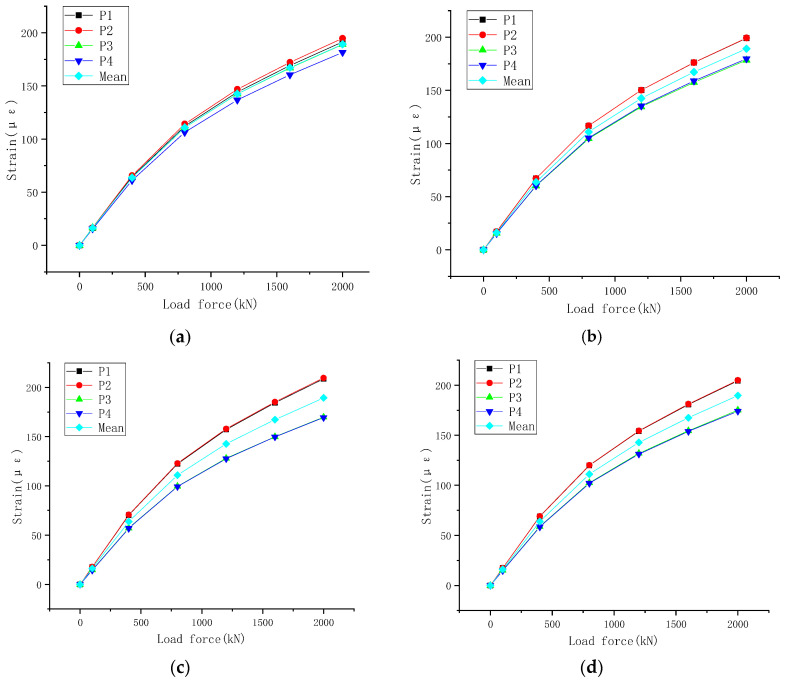
Strain change curve of the welding strain gauge at different corners. (**a**) 0°; (**b**) 90°; (**c**) 180°; (**d**) 270°.

The strain change curves of the welding strain gauge at different corners are depicted in Figure 11. The slopes of these curves remain constant at angles of 90°, 180° and 270°, where there is also an eccentric load. The installation of four welding strain gauges makes it possible to measure the vertical reaction force of the bearing through their average value. Additionally, it allows for the analysis of the eccentric load of the bearing by observing the slope of the strain change curve of a single welding strain gauge. During this test, when the support is rotated by 180°, the deviation from the loading center is the largest. The difference between the strain difference of P2 and P4 and the average strain error is 21.3%.

## 6. Conclusions

In this study, a new type of intelligent pot bearing with pipe-type welding strain gauges was developed for load measurement and temperature compensation. The conclusions are summarized as follows:

(1) A pipe-type spot welding strain gauge comprises a stainless steel tube with a diameter of 1.6 mm welded with a 0.05 mm substrate, and then the resistance strain gauge is threaded into the middle and glued. Using a portable small spot welding machine, it was welded to the pot bearing, which was tested immediately to ensure firmness. This process involves spot welding to the bearing; the voltage is only 3 V to 5 V and will not damage the internal structure of the bearing.
(2) Finite element analysis was conducted on the pot bearing, and the optimal layout of the welded strain gauge on the bearing was obtained. When comparing P1~P4 on the surface of the pot bearing with P5~P8 on the side, P1~P4 on the surface had the best layout.
(3) The calibration test was conducted on the vertical bearing capacity of the bearing compression and shear testing machine at room temperature of the pot-type bearing. Subtractive polynomial cubic polynomial fitting was used, and the curve fitting for each segment was high, with a goodness of fit R^2^ > 0.999.
(4) Temperature compensation tests were carried out in a temperature chamber using the testing machine to correct measurement errors in the welding strain gauge caused by thermal zero drift and thermal sensitivity drift. A method of minimizing the sum of the squares of the lateral distances of coordinates is proposed, which provides a straight line that minimizes the sum of the squares of the lateral distances of each point from the line. The slope of the line is a coefficient, and the calibration formula equation uses the coefficient for temperature compensation. After considering temperature compensation, the measurement error of the intelligent support of the welding strain gauge was reduced to approximately ±1.8%, which is satisfactory.
(5) The research results can be used in the intelligent monitoring of bridges. The pipe-type structure protected the resistance strain gauge. These sensors can be easily replaced with new sensors when repairs are needed. In the future, a set of monitoring system software for an intelligent pot bearing is developed, which can measure the load value of the pot bearing in real time and bridge damage. The software can be applied to the bridge intelligent support monitoring system to carry out remote intelligent monitoring of the support, grasp the dynamic state of the support in real time and a long time, and then monitor the dynamic health of the bridge.

## Figures and Tables

**Table 1:** Ogden model parameters.

$\mu_{1}$	$\alpha_{1}$	$\mu_{2}$	$\alpha_{2}$	$\mu_{3}$	$\alpha_{3}$	d
6.3	1.3	0.012	5.0	−0.1	−2.0	2 × 10^−4^

**Table 2:** Material parameters of the pot rubber bearing [22].

Component	Elastic Modulus (MPa)	Poisson’s Ratio
Support top plate, steel basin, steel lining plate, stainless steel sliding plate	2.05 × 10^5^	0.3
Pressure-bearing rubber sheet	4.4 × 10^3^	0.5
Teflon skateboard	1.5 × 10^3^	0.4

**Table 3:** Load test data.

Test Force Value (kN)	P1~P4 Strain Mean (με)			P5~P8 Strain Mean (με)		
	1st Cycle	2nd Cycle	3rd Cycle	1st Cycle	2nd Cycle	3rd Cycle
0	0.0	0.0	0.0	0.0	0.0	0.0
100	19.8	19.7	19.6	8.0	9.0	7.1
400	70.7	70.8	70.5	32.6	32.4	32.2
800	117.8	117.9	117.6	72.8	72.9	73.1
1200	149.3	149.3	149.5	113.1	113.3	112.9
1600	173.9	173.7	173.9	145.6	145.5	145.1
2000	193.8	193.9	193.7	170.9	171.2	171.1

**Table 4:** Test data under different temperatures.

Temperature (°C)	Test Force Value (kN)	P1~P4 Strain Mean (με)			P5~P8 Strain Mean (με)		
		1st Cycle	2nd Cycle	3rd Cycle	1st Cycle	2nd Cycle	3rd Cycle
−10	0	0.0	0.0	0.0	0.0	0.0	0.0
	100	16.3	16.2	16.2	7.4	8.0	7.2
	400	63.7	63.8	63.7	29.1	28.8	29.0
	800	110.9	110.8	110.6	68.6	68.4	68.5
	1200	142.6	142.7	142.8	110.1	110.4	110.2
	1600	167.2	167.2	167.3	142.3	142.6	142.7
	2000	187.2	187.1	187.2	166.0	166.4	163.9
0	0	69.7	69.9	70.1	47.9	47.8	47.6
	100	86.2	85.9	85.9	54.7	55.7	54.7
	400	133.4	133.5	133.4	76.8	76.5	76.7
	800	180.6	180.5	180.3	116.3	116.1	116.2
	1200	212.3	212.4	212.5	157.8	158.1	157.9
	1600	236.9	236.9	237.2	190.1	190.3	190.4
	2000	256.9	256.8	256.9	213.7	214.1	207.6
10	0	139.1	138.9	138.8	94.6	94.5	94.4
	100	155.7	155.6	155.6	101.7	102.1	102.3
	400	203.1	203.2	203.1	123.5	123.3	123.8
	800	250.3	250.2	250	163.2	163.2	163.1
	1200	282.1	282.1	282.2	204.7	205.3	204.6
	1600	306.6	306.6	306.7	237.4	237.0	236.8
	2000	326.6	326.5	326.6	258.6	260.2	260.3
20	0	206.3	208.1	207.3	141.1	141.3	141.3
	100	224.6	224.6	224.7	148.1	147.1	147.1
	400	272.1	272.2	272.1	168.9	169.2	169.1
	800	319.0	319.2	319.3	208.5	208.7	208.6
	1200	350.2	351.1	351.0	250.5	250.2	250.3
	1600	374.7	375.6	375.7	282.7	282.4	282.8
	2000	395.6	395.5	395.6	306.5	306.1	305.3

**Table 5:** Comparison of the measurement data of welding strain gauges P1~P4.

Standard Pressure Ring Measurements (kN)	Welding Strain Gauge P1~P4 Calculation Force Value (kN)				Average Error (%)
	−10 °C	0 °C	10 °C	20 °C	
100	100.0	102.3	103.8	101.4	1.8
400	399.1	401.3	403.4	400.7	0.28
800	801.9	805.1	808.4	804.3	0.61
1200	1198.8	1203.3	1208.3	1197.3	0.16
1600	1599.8	1606.7	1611.2	1598.5	0.25
2000	2000.5	2007.3	2014.2	2005.6	0.34

**Table 6:** Comparison of the measurement data of welding strain gauges P5~P8.

Standard Pressure Ring Measurements (kN)	Welding Strain Gauge P5~P8 Calculation Force Value (kN)				Average Error (%)
	−10 °C	0 °C	10 °C	20 °C	
100	110.2	120.7	123.8	103.2	14.4
400	394.7	405.4	406.4	391.5	-0.12
800	800.1	808.7	810.3	797.5	0.51
1200	1199.3	1209.0	1210.5	1196.5	0.31
1600	1602.3	1616.5	1616.6	1598.3	0.52
2000	1998.2	1989.8	2012.0	2001.5	0.02

**Table 7:** Test data of different corner welding strain gauges P1~P4.

Angle	Test Force Value	Strain Value (με)				
(°)	(kN)	P1	P2	P3	P4	Mean
0	0	0	0	0	0	0.0
	100	16.4	16.7	16.5	15.6	16.3
	400	64.4	65.6	63.7	61.1	63.7
	800	112.2	114.2	110.8	106.4	110.9
	1200	144.3	146.8	142.5	136.8	142.6
	1600	169.2	172.2	166.9	160.5	167.2
	2000	191.4	194.8	189.0	181.6	189.2
90	0	0	0	0	0	0
	100	17.0	17.1	15.6	15.4	16.2
	400	67.0	67.1	60.1	60.4	63.7
	800	116.7	116.8	104.6	105.3	110.9
	1200	150.2	150.2	134.5	135.4	142.6
	1600	176.1	176.2	157.6	158.9	167.2
	2000	199.2	199.4	178.5	179.8	189.2
180	0	0	0	0	0	0
	100	17.8	17.9	14.8	14.5	16.3
	400	70.2	70.61	57.2	56.9	63.7
	800	122.3	122.9	99.5	99.2	110.9
	1200	157.3	158.0	128.0	127.6	142.7
	1600	184.4	185.3	149.9	149.7	167.3
	2000	208.7	209.7	169.8	169.4	189.4
270	0	0	0	0	0	0
	100	17.5	17.5	15.2	14.9	16.2
	400	68.8	69.0	58.9	58.5	63.8
	800	119.8	120.1	102.5	102.0	111.1
	1200	154.1	154.5	131.9	131.1	142.9
	1600	180.7	181.2	154.5	153.8	167.5
	2000	204.5	205.0	175.1	174.1	189.7

## Data Availability

Data will be made available on request.
